# Prevalence of overweight and obesity among police officers in Riyadh City and risk factors for cardiovascular disease

**DOI:** 10.1186/s12944-017-0467-9

**Published:** 2017-04-14

**Authors:** Abdullah S. Alghamdi, Mohammed A. Yahya, Ghedeir M. Alshammari, Magdi A. Osman

**Affiliations:** 1 Ministry of Inter, P.O BOX 61008, Abaha, Saudi Arabia; 2 Metabolic Disorders Lab, Food Science and Nutrition Department, College of Food and Agricultural Sciences, King Saud University, P.O. Box 2460, Riyadh, 11451 Saudi Arabia

## Abstract

**Background:**

Despite the prevalence of overweight and obesity and increases in associated diseases such as diabetes and heart disease in the Saudi population, no studies have addressed the spread of obesity among Saudi police officers. Therefore, the present study aimed to assess the prevalence of overweight and obesity and associations with biochemical parameters among the police in Riyadh.

**Method:**

The study involved a cross-sectional survey of 160 police officers in Riyadh, Saudi Arabia. Anthropometric measurements, blood pressure, lipid profiles and fasting blood sugar levels were measured for all individuals.

**Results:**

According to the results, the average body mass index (BMI) was 27.5 ± 5.1, indicating an increase in overweight in this population and 66.9% were overweight or obese. Moreover, the mean systolic and diastolic blood pressure values were 119.5 and 79.4 mmHg, respectively, within normal limits. The mean total cholesterol (TC), high-density lipoprotein-cholesterol (HDL-C), low-density lipoprotein-cholesterol (LDL-C), and triglyceride (TG) levels were 187.5, 43.9, 119.5 and 124.5 mg/100 ml, respectively.

**Discussion:**

These BMI and biochemical findings suggest a high proportion of overweight and obese individuals in the sample population, as well as an increase in the proportion of individuals with high levels of biochemical indicators who are therefore susceptible to heart disease and diabetes.

**Conclusion:**

The study recommends using preventive programs to combat obesity and overweight and related diseases and conducting further studies using measures other than BMI.

## Background

Generally, police work is recognized as a dangerous occupation, and the health of police officers must be considered [1]. In all countries, police officers play important roles by ensuring security and stability. These individuals perform specialized work involving exposure to violence, which can affect their health directly or indirectly. Currently employed police personnel have also been reported to have a high prevalence of obesity and related diseases such as hypertension, hyperlipidemia, cigarette smoking and sedentary lifestyle [2].

Police officers generally face increased risks of hyperlipidemia and metabolic syndrome, conditions that contribute to a higher prevalence of cardiovascular disease [3–5]. Epidemiological reports have demonstrated a higher prevalence of obesity among police officers, compared to non-police workers. Therefore, it is important to investigate the associations between body weight and biochemical parameters. Accordingly, treatment and preventive strategies should be implemented to improve the abilities of these officers during work periods [6, 7]. Recently, some.

Obesity is caused by an imbalance between energy intake and energy expenditure [8–10]. One of the most recent global estimates found that roughly 500 million adults are obese. Obesity is a complex condition with serious physical and psychological impacts on overall health. It has been defined by the World Health Organization (WHO) as abnormal or excessive fat accumulation that may impair health. In contrast, overweight is defined as a body mass index (BMI) of ≥25 kg/m^2^. The WHO uses BMI, which is calculated by dividing weight (in kg) by the squared height (in meters), to classify obesity [11]. The many benefits of economic growth are countered by the negative impacts on health, including a poor diet, sedentary lifestyle, and obesity [12, 13]. In the Eastern Mediterranean Region, obesity may cause non-communicable diseases such as diabetes mellitus, cerebral hypertension, cardiovascular disease, various cancers, osteoarthritis, and breathing disorders [14]. Obesity has become a major concern in Saudi Arabia because of these related diseases. Diet and nutrition, physical activity, education, and cultural environment have all been linked to the prevalence of obesity [15]. In an Indian study, most police officers (68%) ranged in age from 25 to 39 years. The prevalence of hypertension in this population was 56.86%, and 43.13% of officers were newly diagnosed. A waist circumference > 90 cm was associated with a higher prevalence of hypertension among police personnel [16].

The prevalence of obesity in Saudi Arabia is approximately 28% in males and 44% in females [17]. Al-Qahtani et al. [18] conducted a study of the obesity prevalence in Saudi adult soldiers, not including police officers. The study involved a cross-sectional survey of 2250 Saudi male soldiers aged 20–60 years who resided in northern Saudi Arabia. Anthropometric measurements, blood pressure, a brief medical history, serum lipid profile, and fasting plasma sugar measurements were requested for all subjects. The authorsobserved prevalence rates of 82%, 32%, and 29% for overweight and obesity, elevated triglyceride levels, and high blood pressure.

To our knowledge, no previous research has addressed the prevalence of overweight and obesity among Saudi police officers and the relationships between lipid profiles and weight in this population. Therefore, the present study aimed to examine the associations between weight and individual biochemistry measurements in Saudi police officers.

## Methods

In this cross-sectional survey, 160 male police officers working in Riyadh city voluntarily agreed to participate in this study. All participants received questionnaires including questions about demographic data such as age, social status, number of family members, monthly income, educational level, military rank, personal history and health, physical activity, smoking status, and types of foods and beverages consumed. These questionnaires were distributed to police stations in Riyadh city, and officers’ body weights were measured by the researchers, who then calculated BMI values to yield units of kg/m^2^. Study participants were divided into four categories according to BMI: <18.5 (underweight), 18.5–24.9 (normal), 25–29.9 (overweight) and ≥30 (obese) [19]. Each participant was then supplied with a requisition for laboratory investigation after an overnight (12-h) fast. The requested laboratory tests included fasting plasma glucose (FPG), serum total cholesterol (TC), triglycerides (TG), low-density lipoprotein-cholesterol (LDL-C), and high-density lipoprotein-cholesterol (HDL-C). Blood pressure was measured after the participant rested in the waiting room for 10–15 min. The measurements were conducted using a standard mercury sphygmomanometer while the subject sat in an office chair with an arm resting on the table. The point of appearance of the first Korotkoff sound was defined as the systolic blood pressure (SBP), whereas the point of disappearance of the last Korotkoff sound indicated the diastolic blood pressure (DBP); measurements were made to the nearest millimeter of mercury (mm Hg).

## Statistical analysis

Data are expressed as means ± standard errors (SEs). Differences between groups were analyzed using a one-way analysis of variance, followed by Duncan’s Multiple Range (DMR) test. SPSS 21.0 software (SPSS Inc., Chicago, IL, USA) was used for the analyses. Differences were considered statistically significant at *P* values < 0.05.

## Results and discussion

Most previous studies that have assessed the prevalence of obesity in the Saudi population have reported varied results, with prevalence rates ranging from 16 to 46% for clinical obesity and from 29 to 35% for overweight. Only one study, conducted by Al-Qahtani et al. [18], has evaluated active service personnel in the armed forces of Saudi Arabia. Our results, shown in Table 1, demonstrate that the police officers in this study ranged in age from 20 to 56 years (mean of age = 34.4 ± 8.3 years). The average weight, height, BMI, systolic blood pressure, and diastolic blood pressure were 79 ± 15.2 kg, 169.6 ± 6.3 cm, 27.5 ± 5.1 kg/m^2^, 119.5 ± 13.9 mmHg, and 79.4 ± 11.9 mmHg, respectively.

**Table 1:** Anthropometric and laboratory values among obese and non-obese participants

Characteristics	Mean ± standard error
Age (years)	34.4 ± 8.3
Weight (kg)	79 ± 15.2
Height(cm)	169.6 ± 6.3
Body mass index (BMI) (kg/m^2^)	27.5 ± 5.1
Systolic blood pressure (mm Hg)	119.5 ± 13.9
Diastolic blood pressure (mm Hg)	79.4 ± 11.9

The distribution of participating police officers into four BMI groups was as follows: underweight, 1.3%; normal, 31.9%; overweight, 42.5%; and obese, 24.4% (Table 2). Subjects with BMI values within the underweight and normal ranges accounted for only 33.2% of the study population. In other words, more than 66.9% of the study subjects were either overweight or obese. These results are similar to those from the study by Al-Qahtani et al. [18] who observed prevalence rates of 43.9% and 81.4% for overweight and obesity among Saudi adult soldiers in northern Saudi Arabia. The prevalence rates of overweight and obesity among police officers in this study may be attributable to poor levels of health and nutritional awareness, increased intake of fatty foods and sugars, and a low level of physical activity. A strong association between obesity and lack of physical activity has been observed among Saudi adolescents [20].

**Table 2:** Distribution of participants according to body mass index (BMI) classification

BMI category	Percentage (%)
Underweight) < 18.5 kg/m^2^)	1.3%
Ideal weight (18.5–25.0 kg/m^2^)	31.9%
Overweight (25.1–29.9 kg/m^2^)	42.5%
Obese (≥30.0 kg/m^2^)	24.4%
Total	100%

Table 3 shows that police officers involved in this study had an average blood sugar level of 94.9 mg/100 ml and average TC, TG, HDL-C and LDL-C levels of 187.5 mg/100 ml, 124.5 mg/100 ml, 43.9 mg/100 ml and 119.5 mg/ml, respectively.

**Table 3:** Biochemical parameters of individuals participating in the study

Biochemical parameter	Value (mean ± standard error)
Blood sugar, mg/100 ml	94.9 ± 8.3
Total cholesterol, mg/100 ml	187.5 ± 32.9
High-density lipoprotein cholesterol, mg/100 ml	43.9 ± 8.6
Low-density lipoprotein cholesterol, mg/100 ml	119.5 ± 24.4
Triglycerides, mg/100 ml	124.5 ± 50.9

The results in Table 4 demonstrate that 37% of participants in this study had a high TC level (≥200 mg/100 ml). Moreover, 34% and 14% had a low level of HDL-C and high level of LDL-C, respectively (<40 mg/100 ml and >150 mg/100 ml, respectively). These results agree with those reported by Al-Nozha et al. [21], who noticed that Saudi males was 54.9% of Saudi men had elevated levels of cholesterol, and by Ogbeide et al. [22], who observed that 43.3% of males in Al-Kharj city had a cholesterol level > 200 mg/100 ml.

**Table 4:** Percent values of some health indicators according to body mass index category

Biochemical parameters	% Of the study sample	Ideal weight	Overweight	Obese
Blood sugar >110 mg/100 ml	12.0%	16.7	25.0	58.3
Total cholesterol >200 mg/100 ml	37.0%	13.5	37.4	48.6
High-density lipoprotein cholesterol <40 mg/100 ml	34.0%	50	23.5	26.5
Low-density lipoprotein cholesterol < 150 mg/100 ml	14.0%	21.4	21.4	57.1
Triglycerides >150 mg/100 ml	20.0%	25.0	20.0	55.0
Systolic blood pressure >130 mmHg	12.0%	00	33.3	66.6
Diastolic blood pressure >90 mmHg	13.0%	00	38.5	61.5

Our study results suggest a positive relationship between blood cholesterol levels and BMI among participants, as 48.6%, 37.8% and only 13.5% of those with high cholesterol were obese, overweight, and normal weight, respectively. In addition, 57.1% and 21.4% of participants with increased LDL-C levels were obese and normal weight, respectively. Among subjects with low HDL-C, 26.5% were obese and −23.5% were overweight. According to Table 4, 20% of participants had a high TG ratio (based on the normal level of TG less than 150 mg/100 ml), a lower rate than that reported by Al-Nozha et al. [21], who observed a high TG rate of 47.6% among adult males in Saudi Arabia. In addition, a previous study found that TG values increased with increasing BMI; normal-weight individuals had an average TG level of 110 mg/100 ml, whereas overweight and obese individuals had levels of 132 mg/100 ml and 142.3 mg/100 ml, respectively [23]. Generally, the high levels of TG and cholesterol observed among police officers in our study may be due to the increased prevalence of overweight and obesity. Many previous studies have indicated a close link between obesity and increased cholesterol levels, possibly as a result of low LDL receptor activity levels and genetic factors, in addition to an increase in the consumption of saturated fats [24]. Poor health behaviors, which usually include a high intake of red meat, fried foods, and prepared food from restaurants, low intake of fiber-rich foods, and physical inactivity, may be the cause of the high blood lipid levels observed in police officers in the present study. In addition to the preceding factors, the requirement for night-shift security maintenance and related stressors could lead to increases in TG and cholesterol levels. This was previously noted by Azhari [25] who, in a study of employees in Jeddah city, observed increases in blood lipid levels among those who worked during the night shift, compared with those who worked during the day shift. A high blood cholesterol level is a risk factor for coronary heart blockage and consequent angina [26]. Therefore, obese individuals in this study, who have cholesterol levels that exceed normal limits, are susceptible to angina along with other risk factors that lead to disease such as hypertension, stress and physical inactivity. We additionally note that the majority of these factors were distributed among obese individuals in our sample.

On the other hand, the results in Table 4 indicate that 12% of participants exhibited notably elevated blood sugar levels (>110 mg/100 ml); this result was higher than the 5.7% reported by Karim et al. [27] for adult males in a Saudi Arabian population. Increased body weight leads to elevated blood sugar levels; the study found that among participants with high blood sugar levels, approximately 58.3% were obese and 25% were overweight. Blood sugar levels increased with increasing BMI, as the rates among normal-weight, overweight, and obese participants were 6.1%, 7.7%, and 25%, respectively (Table 4). These results agree with those obtained by Al-Qahtani et al. [28] and Timar et al. [29], who reported a high blood sugar prevalence of 2.9% among normal-weight individuals compared to 4.8% and 5.8% among overweight and obese individuals, respectively. The increased blood sugar levels observed in obese police officers may be attributable to several causes, including an increased intake of sugars and sweets and low intake of fiber-rich foods; notably, our analysis determined that 40.6% of participants never consumed brown bread, and more than half did not eat vegetables (fresh or cooked) on a daily basis. Other factors may also be attributable, such as a decreased insulin sensitivity, which is often associated with obesity and is a direct cause of high blood sugar levels. Christopher et al. [26] noted that overweight and obesity led to poor insulin sensitivity and was associated with increased blood sugar levels, increased intake of carbohydrate-rich foods, and a lack of physical activity [30]. Previous studies show benefits to use specific food to reduce risk factors of obesity and its related disease. For instant, the nutraceuticals ingredients are beneficial to vascular health may represent useful compounds that are able to reduce the overall cardiovascular risk induced by dyslipidemia [31]. As a result, we suggest to involve a functional food in food planning for police officers and another workers.

## Conclusion

This study observed a high prevalence of overweight and obesity among Saudi police officers in Riyadh city. A comprehensive study of the factors that lead to the prevalence of obesity among Saudi police officers is needed, and preventive and remedial plans should be developed to protect these officers from obesity and associated complications.

## Abbreviations

BMI: Body mass index
DBP: Diastolic blood pressure
DMR: Duncan’s Multiple Range
FPG: Fasting plasma glucose
HDL-C: High-density lipoprotein-cholesterol
LDL-C: Low-density lipoprotein-cholesterol
SBP: Systolic blood pressure
SE: Standard error
TC: Total cholesterol (TC),
TG: Triglycerides
WHO: World Health Organization
